# Pediatric tertiary emergency care departments in Zagreb, Rijeka, and Split before and during the coronavirus disease 2019 pandemic: a Croatian national multicenter study

**DOI:** 10.3325/cmj.2021.62.580

**Published:** 2021-12

**Authors:** Ante Šokota, Laura Prtorić, Iva Hojsak, Ivana Trivić, Filip Jurić, Kristina Lah, Tomulić, Jelena Roganović, Harry Nikolić, Ana Bosak Veršić, Joško Markić, Marijan Batinić, Goran Tešović

**Affiliations:** 1 Dr. Fran Mihaljević University Hospital for Infectious Diseases, Zagreb, Croatia; 2 Department of Pediatrics, Children's Hospital Zagreb, Zagreb, Croatia; 3 University of Zagreb School of Medicine, Zagreb, Croatia; 4 School of Medicine, J. J. Strossmayer University of Osijek, Osijek, Croatia; 5 Department of Pediatric Surgery, Children's Hospital Zagreb, Zagreb, Croatia; 6 Department of Pediatrics, Clinical Hospital Center Rijeka, Rijeka, Croatia; 7 School of Medicine, University of Rijeka, Rijeka, Croatia; 8 Department of Pediatric Surgery, Clinical Hospital Center Rijeka, Rijeka, Croatia; 9 Department of Pediatrics, University Hospital Center Split, Split, Croatia; 10 School of Medicine, University of Split, Split, Croatia; The first two authors contributed equally.

## Abstract

**Aim:**

To assess the number of visits to pediatric emergency departments in Croatia and reasons for visiting before and during the coronavirus disease 2019 (COVID-19) pandemic.

**Methods:**

We reviewed the medical records of pediatric patients visiting emergency departments of four tertiary medical centers between February 25 and April 25, 2018 and 2019, and between February 25 and April 24, 2020. Antimicrobial prescription was analyzed as well.

**Results:**

There were altogether 46 544 visits – 18218 in 2018, 19699 in 2019, and 8634 in 2020. The overall number of visits in 2020 significantly decreased compared with 2018 and 2019 (52% and 56% reduction, respectively), mostly due to a decreased number of visits due to certain infectious diseases: acute gastroenteritis (89.2%), sepsis/bacteremia (81.2%), urinary tract infections (55.3%), and lower respiratory tract infections (58%). Most visits were self-referrals regardless of the analyzed period, and the majority of patients did not require hospitalization. There were no significant differences in the number of visits requiring urgent medical care, such as those due to seizures and urgent surgery. The most frequently prescribed antibiotic in all periods was amoxicillin, followed by amoxicillin/clavulanate and oral cephalosporins.

**Conclusion:**

A significant reduction in the number of pediatric emergency department visits and hospital admissions is indirectly related to the COVID-19 pandemic. Most of the reduction was due to a decreased number of infectious disease cases. However, the number of visits requiring urgent medical intervention did not change.

The emergence of a new coronavirus disease in Wuhan Province, China, in 2019 marked the first step in the now fully developed and ongoing pandemic. Coronavirus disease 2019 (COVID-19) has profoundly changed our everyday life – from the way we do our shopping to the way we visit the emergency department (ED) (1). The first Croatian cases were reported on February 25, 2020. The initial months after the World Health Organization declared the pandemic (2) were characterized by movement restrictions, resulting in general unease worldwide. As the number of severe COVID-19 cases increased, global health care systems showed their unpreparedness for a global pandemic. The effect of COVID-19 on most medical institutions was almost debilitating (3-14). As the pandemic spread through Europe, most EDs faced the same problem – how to treat their patients while protecting the health of their staff.

Unlike adults, the majority of children with COVID-19 are asymptomatic or have a mild flu-like illness, with limited reports suggesting serious complications in those with preexisting conditions (11,15-17). However, not even children are completely unscathed, with the rise of post-COVID-19 immunological complications, such as multisystem inflammatory syndrome (17,18). The majority of visits to the physician’s office are often due to upper respiratory tract infections, so a global pandemic caused by a respiratory virus should overwhelm EDs (19,20). Reports from across the globe suggest otherwise (4,5,9,10,21). Both adult and pediatric EDs faced declines in the number of visits long before the rise in COVID-19 cases, with the latter exhibiting a sharper slope curve, especially in certain age groups (19,21,22). Having all this in mind, the aim of this study was to investigate the change in the EDs' workload between the pre-COVID-19 periods and a COVID-19 period in four tertiary-level pediatric EDs in Croatia.

## Patients and methods

We retrospectively reviewed the medical records of children aged 0-18 years visiting pediatric EDs during three 60-day periods: February 25-April 25, 2018 and 2019 (pre-COVID years), and February 25-April 24, 2020 (the first COVID-19 pandemic wave). The study was conducted in four Croatian tertiary health care institutions – Dr Fran Mihaljević University Hospital for Infectious Diseases Zagreb (UHID), Children's Hospital Zagreb (CHZ), University Hospital Center Split (UHC Split), and Clinical Hospital Center Rijeka (CHC Rijeka). To gather the data, a CroPedCOVID study group was formed of pediatric, pediatric surgery, and pediatric infectious diseases residents/specialists. The following data were gathered from preexisting electronic (UHID, CHZ, CHC Rijeka) and electronic/paper medical records (UHC Split): sex, date of birth, place of residence, number of household members/siblings, attending daycare/school, referral, preexisting chronic diseases, ICD-10 diagnosis, prescription and duration of antimicrobial therapy if prescribed, and outcome of the visit. The collected data were used to form a database in MS Excel, while grouping the visits into those related to surgical, infectious, and non-infectious conditions. A visit was defined as an individual medical record of a patient regardless of the previous ones, meaning that one patient could have had multiple visits during the same period. The referral type of the visit was categorized as that from a primary physician or another health care setting (secondary/tertiary health care institutions), or self-referral, meaning that the patient’s parents/guardians decided to visit the EDs on their own accord. The general outcome of the visit was defined as hospitalization, urgent surgery, hospital day care, referral to another health care institution, or discharge home. The study protocol was approved by the Ethics Committee of each participating center.

### Outcome measures

The primary outcome was the difference in the number of ED visits between the pre-COVID-19 periods and the early COVID-19 period in all the studied centers and in each studied center separately. The secondary outcomes were the difference between the periods in the referral type, visit outcomes, antibiotic prescription, ICD-10 diagnosis, and the hospitalization rate for specific diagnosis and urgent surgery.

### Statistical analysis

The normality of distribution of non-categorical variables was assessed with the Shapiro-Wilk test. The differences between categorical variables were assessed with the χ^2^ test. ANOVA test was used to assess the difference between the groups. Poisson distribution analysis was performed for count variables (number of patients per day), revealing that the variance was not equal to the mean and that variables were over-dispersed. Therefore, negative binomial regression was used to compare the number of patients per day and the percentage change between 2020 and the pre-COVID-19 periods. The statistical significance was expressed as 95% confidence interval (CI) or *P* value. Statistical analysis was performed with SPSS, version 23.0 (IMB Corp. Armonk, NY, USA).

## Results

### Descriptive statistics

Over the three periods there were altogether 46 544 visits: 9364 (20%) in the UHID, 18 527 (40%) in CHZ, 9071 (19%) in UHC Split, and 9582 (21%) in CHC Rijeka. Overall, 25 127 (54%) patients were seen by pediatric or infectious disease specialists/residents and 21 417 (46%) by pediatric surgeons/surgery residents.

The mean patients' age was 7.8 years (standard deviation [SD] 5.3); 25 800 (55.4%) were male. Similar sex and age distribution was observed in all the studied periods. In 2018, there were 10 254 (56.3%) male participants, 10 760 (54.6%) in 2019, and 4785 (55.4%) in 2020. The number of visits in 2018 was 18 218, and the number of visits in 2019 was 19 699. In 2020, this number significantly decreased, to only 8634 visits, which represents 52% and 56% reduction compared with 2018 and 2019, respectively (*P* < 0.0001 for both).

### Outcomes

**Figure 1:**
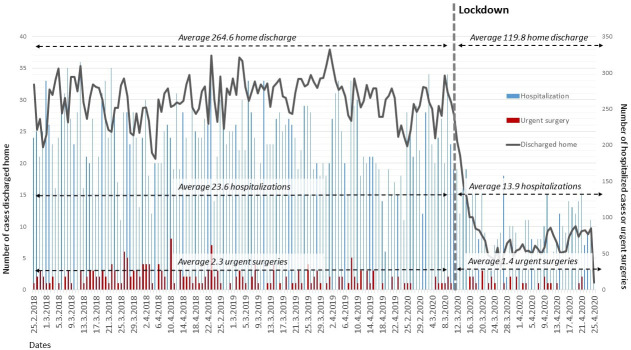
The number of patients who were discharged home without admission to the hospital (right axis) and of those who were hospitalized or referred to urgent surgery (left axis) during the studied periods and change after the lockdown (March 11, 2020); average presented as mean/day.

In all centers, the number of visits significantly decreased, with the greatest reduction being observed in UHID (-71.6%; 95% CI -79.3 to -61.0%). The majority of patients in all periods were self-referred. A significant decrease in 2020 compared with the pre-COVID-19 years was observed regardless of the referral type (Table 1). In all the studied periods, most patients were discharged home without being hospitalized (Figure 1). The total number of hospitalizations during the COVID-19 period significantly decreased, while the number of urgent surgeries remained unchanged (Table 1).

**Table 1:** Difference in the total number of visits, the number of visits per hospital, type of referral, outcome of the emergency department visit, and antibiotic prescription between three periods (2018, 2019, 2020) and percentage change in 2020 compared with 2018 and 2019

	Mean per day (95% CI*)			Mean difference, mean per day (95% CI)		Percent change 2020 with respect to 2018 and 2019 (95% CI)
	2018	2019	2020	2020 to 2018	2020 to 2019	
Total number of visits	303.6 (235.7-391.2)	328.2 (254.7-422.9)	141.5 (110-182.1)	-162.1 (-246.9 to -77.3)	-162.1 (-246.9 to -77.3)	-55.2 (-67.1 to -39)
University Hospital for Infectious Diseases	66.1 (51.1-85.5)	71.5 (55.4-92.3)	19.5 (15.1-25.3)	-46.6 (-64.3 to -28.8)	-52 (-70.9 to -33.1)	-71.6 (-79.3 to -61)
Children’s Hospital Zagreb	120.9 (93.8-155.9)	126.9 (98.4-163.5)	61 (47.3-78.7)	-59.9 (-94.3 to -25.5)	-65.8 (-101.6 to -30)	-50.7 (-63.9 to -32.7)
Split University Hospital Center	57.8 (44.8-74.6)	63.4 (49.1-81.8)	29.5 (22.8-38.1)	-28.3 (-44.9 to -11.8)	-33.9 (-51.7 to -16)	-51.3 (-64.4 to -33.5)
Rijeka Clinical Hospital Center	59.9 (46.4-77.3)	66.4 (51.5-85.7)	33.4 (25.8-43.1)	-26.6 (-44.1 to -9)	-33.1 (-52.1 to -14.1)	-47.2 (-61.4 to -27.8)
Pediatric emergency department	164.7 (127.8-212.3)	181.2 (140.6-233.5)	71.8 (55.7-92.4)	-92.9 (-138.5 to -47.4)	-109.4 (-158.8 to -60)	-58.5 (-69.6 to -43.4)
Pediatric surgery emergency department	139 (107.8-179.9)	147.1 (114.1-189.6)	69.8 (54.2-89.9)	-69.2 (-108.6 to -29.7)	-77.3 (-118.6 to -36)	-51.2 (-64.2 to -33.5)
Self-referral	229.9 (178.4-296.3)	259.5 (201.4-334.4)	108 (84-139)	-121.9 (-186.3 to -57.5)	-151.5 (-222.7 to -80.3)	-55.9 (67.6 to -39.9)
Referred by general practitioner	32.6 (25.2-42.1)	29.1 (22.5-37.6)	15.7 (12.1-20.3)	-17 (-26.3 to -7.6)	-13.4 (-21.9 to -4.9)	-49.2 (-63.1 to -30.2)
Hospitalizations	24.2 (18.7-31.3)	23 (17.7-29.7)	13.9 (10.7-18)	-10.3 (-17.5 to -3.1)	-9.1 (-16 to -2.2)	-41.2 (-61.4 to -27.8)
Urgent surgery	2.5 (1.8-3.5)	1.9 (1.3-2.8)	1.4 (0.9-2.3)	-1.1 (-2.2 to -0.04)	-0.5 (-1.5 to 0.5)	-37.7 (-64.1 to 8)
Discharged home	256 (198.6-329.8)	273.3 (212.1-352.1)	119.8 (93.1-154.2)	-136.1 (-207.7 to -64.6)	-153.4 (-229 to -77.9)	-54.7 (-66.8 to -38.3)
Antibiotic prescription	38 (29.4-49.1)	32.6 (25.2-42.1)	16 (12.3-20.7)	-22 (-32.6 to -11.4)	-16.6 (-26 to -7.3)	-54.7 (-67 to -37.9)

*CI – confidence interval.

In 2020, the number of visits significantly decreased in the majority of diagnosis categories, especially in the category of infectious diseases (Table 2). The number of visits due to acute gastroenteritis (-89.2%, 95% CI -92.5 to -84.3%) and sepsis/bacteremia (-81.2%, 95% CI -89.3 to -67.0%) notably decreased, while the number of visits due to infectious mononucleosis and exanthema subitum decreased insignificantly. Although urinary tract infections (UTIs) are not a communicable infectious disease, the number of visits due to this type of infection decreased significantly, with no significant difference regarding age, sex, or daycare attendance in all the observed periods (Table 2). Hospitalization rates for lower respiratory tract infections (LRTI) and UTIs significantly decreased, while those for varicella, influenza, and unspecified fever remained unchanged (Table 2). Among visits due to LRTIs (n = 2183), 334 (15.3%) were due to acute bronchiolitis, which represented a non-significant decrease compared with the pre-COVID-19 period (-20.9%; 95% CI -44.5%-12.8%). Acute bronchitis was diagnosed in 903 (41.4%) and pneumonia in 852 (39%) patients who visited due to LRTIs, both rates significantly decreasing in 2020 (-45.7%; 95% CI -63.3 to -19.8% and -32.4%; 95% CI -54 to -7%, respectively). There was a decrease in the number of visits due to non-infectious diseases, such as abdominal pain, chest pain, and headache (Table 2). The visits due to different traumas significantly decreased, but those for trauma requiring hospitalization or urgent surgery remained unchanged (Table 2). Additionally, there was no difference in the number of visits due to seizures and acute appendicitis (Table 2).

**Table 2:** Difference in the number of cases diagnosis categories per day between three periods (2018, 2019, 2020) and percentage change in 2020 compared with 2018 and 2019

	Mean per day (95% CI*)				Mean difference, mean per day (95% CI)			Percent change 2020 with respect to 2018 and 2019 (95% CI)
	2018	2019		2020	2020 to 2018	2020 to 2019		
Infectious diseases								
Acute gastroenteritis	12.1 (9.3-15.7)	17 (13.1-22.1)	2.8 (1.9-4.2)		-9.2 (-12.6 to -5.9)	-16.6 (-18.8 to -9.6)	-89.2 (-92.5 to -84.3)	
Upper respiratory tract infections	40.1 (31-51.8)	48.2 (37.3-62.2)	16.2 (12.5-21)		-23.9 (-35 to -12.8)	-32 (-45 to -18.9)	-63.9 (-73.7 to - 50.5)	
Lower respiratory tract infections	13.5 (10.4-17.6)	16.5 (12.7-21.3)	7.2 (5.4-9.7)		-6.3 (-10.4 to -2.2)	-9.2 (-14 to -4.4)	-58 (-69.7 to -41.7)	
Lower respiratory tract infections requiring hospitalization	3.2 (2.4-4.2)	3.4 (2.5-4.5)	1.4 (1-2)		-1.7 (-2.8 to -0.7)	-1.9 (-3 to -0.8)	-55.7 (-69.9 to -34.9)	
Sepsis and bacteremia	1.7 (1.1-2.6)	2.4 (1.8-3.3)	1.3 (0.6-2.5)		-0.4 (-1.5 to 0.6)	-1.1 (-2.3 to 0.01)	-81.2 (-89.3 to -67)	
Varicella	1.6 (1.1-2.2)	1.8 (1.3-2.4)	0.2 (0.1-0.3)		-1.4 (-1.9 to -0.9)	-1.6 (-2.1 to -1)	-89.1 (-94.5 to -78.5)	
Varicella requiring hospitalization	1.2 (0.4-3.9)	1.0 (0.3-4.0)	1.0 (0.1-15.9)		-0.2 (-3.3 to 2.9)	0 (-3.1 to 3.1)	-10 (-95.1 to 156)	
Infectious mononucleosis	2.4 (1.8-3.4)	2.9 (2.1-3.9)	1.3 (0.6-2.8)		-1.0 (-2.4 to 0.2)	-1.5 (-2.8 to -0.2)	-49.9 (-77 to 9.2)	
Exanthema subitum	1.3 (0.7-2.5)	1.5 (0.8-2.9)	1.0 (0.2-5.0)		-0.3 (-2.1 to 1.5)	-0.5 (-2.4 to 1.4)	-29.8 (-86.7 to 269.7)	
Influenza	4.9 (3.7-6.5)	4.6 (3.5-6.1)	2.7 (2-3.6)		-2.3 (-3.9 to -0.7)	-1.9 (-3.4 to -0.4)	-44.3 (-60.9 to -20.6)	
Influenza requiring hospitalization	0.2 (0.1-0.3)	0.2 (0.1-0.4)	0.1 (0.1-0.3)		-0.1 (-0.2 to 0.1)	-0.1 (-0.3 to 0.1)	-42.6 (-76.6 to 40)	
Urinary tract infection	3.2 (2.4-4.4)	3.9 (2.9-5.1)	1.6 (1.2-2.2)		-1.7 (-2.7 to -0.6)	-2.3 (-3.5 to -1.1)	-55.3 (-69.4 to -34.6)	
Urinary tract infection requiring hospitalization	0.8 (0.6-1.2)	0.8 (0.6-1.2)	0.4 (0.2-0.6)		-0.4 (-0.8 to -0.1)	-0.4 (-0.8 to -0.1)	-52.3 (-72.3 to -18)	
Unspecified fever	22.1 (17-28.9)	17.3 (13.3-22.5)	11.1 (8.6-14.4)		-11 (-17.4 to -4.5)	-6.2 (-11.6 to -0.8)	-43.6 (-59 to -22.3)	
Unspecified fever requiring hospitalization	1.4 (1-1.9)	0.8 (0.5-1.1)	1 (0.7-1.4)		-0.4 (-1 to 0.2)	0.2 (-0.3 to 0.7)	-9.3 (-41.4 to 40.2)	
Non-infectious diseases								
Seizures	1.9 (1.4-2.6)	2.1 (1.5-2.8)	1.6 (1.1-2.2)		-0.4 (-1.1 to 0.4)	-0.5 (-1.3 to 0.3)	-21.6 (-46.9 to 15.6)	
Seizures requiring hospitalization	1.5 (1.1-2.1)	1.7 (1.3-2.4)	1.1 (0.8-1.6)		-0.4 (-1 to 0.2)	-0.6 (-1.3 to 0.1)	-30.7 (-54.2 to 4.6)	
Headache	2.7 (2-3.6)	3.3 (2.5-4.4)	1.5 (1.1-2)		-1.2 (-2.1 to -0.3)	-1.9 (-2.9 to -0.8)	-50.8 (-66.5 to -27.7)	
Headache requiring hospitalization	0.7 (0.4-1)	0.6 (0.4-0.9)	0.3 (0.2-0.6)		-0.3 (-0.6 to -0.01)	-0.3 (-0.6 to 0.05)	-44.6 (-69.4 to -3.6)	
Abdominal pain	27.5 (21.2-35.6)	25.5 (19.7-32.9)	11.3 (8.7-14.7)		-16.1 (-23.8 to -8.5)	-14.1 (-21.3 to -6.9)	-57.3 (-68.9 to -41.2)	
Abdominal pain requiring hospitalization	3.3 (2.5-4.4)	2.9 (2.1-3.8)	1.5 (1.1-2)		-1.9 (-2.9 to -0.8)	-1.4 (-2.4 to -0.4)	-52.4 (-67.6 to -30.1)	
Abdominal pain requiring urgent surgery	0.3 (0.2-0.5)	0.2 (0.1-0.3)	0.1 (0.02-0.2)		-0.2 (-0.3 to -0.1)	-0.1 (-0.3 to 0.01)	-13.8 (-80.5 to 280)	
Chest pain	2.1 (1.6-2.9)	2.6 (1.9-3.5)	0.7 (0.5-1.1)		-1.4 (-2.1 to -0.7)	-1.8 (-2.6 to -1)	-67.8 (-79.2 to -50.1)	
Chest pain requiring hospitalization	0.1 (0.1-0.3)	0.1 (0.04-0.2)	0.1 (0.02-0.2)		-0.1 (-0.2 to 0.1)	-0.03 (-0.1 to 0.1)	-39.5 (-81.1 to 93.5)	
Appendicitis	0.6 (0.4-0.9)	0.7 (0.5-1.1)	0.5 (0.3-0.7)		-0.1 (-0.4 to 0.1)	-0.3 (-0.6 to 0.1)	-30.3 (-59 to 18.4)	
Trauma	102.6 (79.6-132.4)	109.7 (85.1-141.4)	52.1 (40.4-67.1)		-50.6 (-79.8 to -21.3)	-57.6 (-88.4 to -26.7)	-50.9 (-64 to -33.1)	
Trauma requiring hospitalization	4 (3-5.3)	4.1 (3.1-5.4)	3.1 (2.3-4.1)		-0.9 (-2.3 to 0.6)	-1 (-2.5 to 0.4)	-23.6 (-46.2 to 8.6)	
Trauma requiring urgent surgery	0.8 (0.5-1.1)	0.3 (0.2-0.5)	0.3 (0.2-0.6)		-0.4 (-0.8 to -0.1)	0.02 (-0.2 to 0.3)	-16 (-60.7 to 79.2)	

*CI – confidence interval.

The information about antibiotic prescription was available for 5212 (11.2%) visits. The most frequently prescribed oral antibiotic was amoxicillin (n = 1160; 22.3%), followed by amoxicillin/clavulanate (n = 903, 17.3%), oral cephalosporins (n = 885, 17%), and azithromycin (n = 426, 8.2%). The structure of antibiotic prescribing remained unchanged during all the observed periods. Ceftriaxone was the most frequently used intravenous antibiotic (n = 1060, 20.3%), either as a monotherapy (n = 320 visits, 30.2%) or before switching to an oral antibiotic (n = 740 visits, 69.8%). The hospital with the greatest number of antibiotic prescriptions was UHID (n = 2073, 39.8%), followed by UHC Split (n = 1174, 22.5%), CHZ (n = 1042, 20%), and CHC Rijeka (n = 923, 17.7%). Oral antibiotic prescription significantly differed between the centers (Table 3); amoxicillin was the most frequently prescribed oral antibiotic in UHID and amoxicillin/clavulanate in all other centers.

**Table 3:** Oral antibiotic prescription per hospital

Antibiotic, n (%)	University Hospital for Infectious Diseases (n = 2073)	Children’s Hospital Zagreb (n = 1042)	Split University Hospital Center (n = 1174)	Rijeka Clinical Hospital Center (n = 923)	p
Amoxicillin	902 (43.5)	189 (18.1)	16 (1.4)	53 (5.7)	<0.001
Amoxicillin and clavulanic acid	48 (2.3)	235 (22.6)	287 (24.4)	333 (36.1)	<0.001
Oral cephalosporins	219 (10.6)	203 (19.5)	224 (19.1)	239 (25.9)	<0.001
Azithromycin	71 (3.4)	74 (7.1)	174 (14.8)	107 (11.6)	<0.001

## Discussion

The current study confirms our hypothesis – during the analyzed period of the COVID-19 pandemic the number of emergency visits in all four Croatian centers significantly decreased compared with the pre-COVID years. Other studies worldwide also observed a decreased number of non-COVID-19 related ED visits. This decrease might be explained by a direct/indirect effect of countermeasures and the fear of contracting COVID-19 in hospital (2,5,8-11,13,18,21-26). The most affected center was UHID, with a staggering 71.6% decrease, while other centers had close to 50% decrease (Table 1). This reduction was expected as it was predominantly the result of a decreasing number of infectious disease cases. It seems that strict government-imposed movement restrictions, the closing of schools and daycare centers, increased hand hygiene, and obligatory facemasks wearing, lowered the transmission of not only SARS-CoV-2 but of other communicable infectious diseases, which otherwise overwhelm EDs, especially acute gastroenteritis and LRTIs. Interestingly, although not being a communicable infectious disease, the number of UTI-related visits also decreased, with no significant difference in the incidence regarding age, sex, or daycare attendance. Reasons for this decrease are yet to be elucidated. A possible hypothesis is that “stay-at-home” measures could have led to better hygiene habits, such as more frequent diaper changes, which could have prevented UTIs among toddlers (27,28). Literature review found no research on this topic.

Severe bacterial infections such as sepsis/bacteremia were also in decline. A large part of bacteremia cases in children are pneumococcal infections originating from the upper respiratory tract. Therefore, it seems logical that a limited pneumococcus transmission upon closing of daycare facilities would also reduce the spread of invasive diseases such as occult bacteremia, especially in children under five years (29,30). Though presumably a reduction in occult bacteremia could be a consequence of the introduction of 10-valent pneumococcal conjugate vaccine into the Croatian vaccination schedule in 2019, it is too early to observe the vaccination effect.

Regarding other more common children’s infectious diseases, visits due to varicella decreased as expected, while hospitalizations due to varicella and its complications remained unchanged. This might be explained by the fact that most common varicella complication, soft tissue infection, is associated with self-inoculation of resident bacteria rather than interpersonal transmission (31,32). The stagnation in the number of cases of exanthema subitum or infectious mononucleosis is not surprising, as the sources of infections are usually healthy adults, who acquired the causative viruses at an earlier age (33,34).

While ED visits due to infectious diseases decreased substantially, the number of visits due to non-communicable diseases varied depending on the disease. However, the number of visits requiring urgent medical intervention remained unchanged. This study showed no reduction in the number of visits due to appendicitis nor delves into the etiology of the disease. Numerous other studies reported fewer but more complicated cases of appendicitis, which reignited theories of multifactorial etiology (35-38). Fewer visits were due to abdominal pain, although this did not influence the number of hospitalizations, suggesting that the cause was often banal. The reduction in trauma- or seizure-related visits observed in other countries was not supported by our study (7,23,39-46). Regarding life-threatening conditions, there was no increased mortality although official reports are yet to be expected (35,36,38).

While this study did not assess the impact of COVID-19 on mental health, there were fewer visits due to common nonspecific symptoms, such as headache, chest, or abdominal pain. This finding opens the question of the psychological origin of some non-specific symptoms. Ravens-Sieberer et al (47) reported substantial psychosomatic symptoms in the 11-17 age group two months after the lockdown. Contrary to this, at the very beginning of the pandemic, Liu et al (48) observed a low incidence of somatization in primary-school students. These contradictory results could be explained by the duration of the pandemic and restrictive epidemiological measures. They could suggest the existence of a specific period required to get accustomed to the “new normal,” where the initial fear of COVID-19 overcomes the previous psychosomatic symptoms until the new environment conditions become part of everyday life (47-51).

A secondary outcome of this study was to assess the antibiotic prescription in the studied centers. The most frequently prescribed antibiotic was amoxicillin, with UHID being the most frequent prescriber. The most frequently prescribed antibiotic in the other centers was amoxicillin/clavulanate, followed by oral cephalosporins and azithromycin. These data correspond to local antimicrobial resistance – eg, centers with a high prescription rate of azithromycin have more azithromycin- resistant group A *Streptococcus* (up to 15%), which is still in an upward trend (52,53). Increasing prescription trends of amoxicillin/clavulanate, azithromycin, and second/third generation cephalosporins in Croatia have been observed for more than 15 years, with limited improvement (54-56). This is supported by our study, where oral cephalosporins accounted to 17% of all prescribed antibiotics, with 85% of them belonging to the second and third generation. These observations support the need for national guidelines on antibiotic treatment and better antimicrobial stewardship (27,56-61).

Even though not directly connected to the aim of this study, it is noteworthy that the analyzed EDs belong to the tertiary health care system. Given that most visits were self-referrals not requiring hospitalization or antibiotic treatment and that their incidence significantly decreased during the COVID-19 pandemic, one could wonder if Croatian health care utilization should be better organized. Solutions for the overuse of tertiary health care system are needed.

This study has several limitations: although our centers cover the most of pediatric population in Croatia, data from certain regions that do not gravitate toward our centers were not included (62,63). Furthermore, not all required data were obtained; the majority of data regarding daycare/school attendance and the number of household members were lacking, especially in surgical patient histories as this type of information is not usually collected. Antibiotic prescription rather than antibiotic consumption was assessed, and adherence to the prescribed antibiotic regimen was not investigated. Albeit this could suggest a discrepancy in the actual antibiotic use, official reports of the Agency for Medicinal Products and Medical Devices on antibiotic utilization support our findings (63). Data on antibiotic prescription were collected in 11.2% of visits, and although this seems like a low percentage, it is noteworthy that not all conditions require antibiotic treatment. Some diagnoses could not be further differentiated, for example seizures as an epilepsy symptom or febrile convulsion or sepsis vs bacteriemia, as well as probable vs confirmed cases of influenza, mostly due to a lack of adequate archiving techniques. This study did not involve institutions dealing with mental health issues in children, so the impact of COVID-19 on mental health could not be assessed. Finally, the pandemic is still ongoing, so the data are not complete. However, we believe that the studied time periods represent a credible sample for the evaluation of the impact of COVID-19. This is especially the case given that the restrictions in the first 60 days of the Croatian epidemic were much more rigorous than in the rest of the epidemic period. In conclusion, this is the first national study evaluating the impact of COVID-19 pandemic on pediatric EDs in Croatia. Although data clearly showed a significant decrease in the number of ED visits, further studies are needed to determine the reasons for the decrease in both infectious and non-infectious diseases. Lessons learned during this pandemic should make their way into the routine practice.
